# Patient Initiated Follow-Up (PIFU): how can rheumatology departments start to reap the benefits? A consensus document

**DOI:** 10.1093/rap/rkae091

**Published:** 2024-08-29

**Authors:** Raj Sengupta, Marwan Bukhari, Zoe Cole, Stuart Kyle, Gordon MacDonald, Kirsten McKay, Anushka Irani, Mark Perry

**Affiliations:** Royal National Hospital for Rheumatic Diseases, Royal United Hospitals, Bath, UK; Centre for Therapeutic Innovation, University of Bath, Bath, UK; Department of Rheumatology, Royal Lancaster Infirmary, Lancaster, UK; Department of Health and Sciences, Lancaster University, Lancaster, UK; Department of Rheumatology, Salisbury District Hospital, Salisbury, UK; Department of Rheumatology, Royal Devon University Hospital NHS Foundation Trust, North Devon District Hospital, Barnstaple, UK; Department of Rheumatology, Royal Berkshire NHS Foundation Trust, Reading, UK; Department of Rheumatology, South Devon Healthcare NHS Foundation Trust, Torbay Hospital, Torquay, UK; Mayo Clinic, Jacksonville, FL, USA; Department of Rheumatology, Derriford Hospital, Plymouth, UK

**Keywords:** PIFU, digital, rheumatology, remote monitoring, ePROMs, reducing backlog, patient safety, patient empowerment, active monitoring, escalation, personalization

## Abstract

Patient Initiated Follow-Up (PIFU) is gaining momentum in the NHS, aiming to optimize outpatient care amidst rising service demands. PIFU is valuable in rheumatology, where the increasing demand for ongoing management exacerbates the patient backlog. Importantly, PIFU has demonstrated comparable safety and outcomes to traditional care in numerous studies. PIFU empowers patients, drives personalized care, increases efficiency, and has the potential to reduce waiting lists by allowing services to focus on new and acute cases. Effective PIFU implementation includes careful selection of patients, educating patients and healthcare staff, well defined operational guidelines, and robust remote monitoring. Digital solutions can enhance PIFU through patient education, active remote monitoring and streamlined escalation. Electronic Patient Reported Outcome Measures (ePROMs) provide a suitable and safe metric to monitor patients remotely. Given the potential benefits, outpatient departments should consider investing in PIFU as a solution to current healthcare delivery challenges and as a means for future proofing clinical systems against increasing service demands.

Key messages

- PIFU empowers patients, increases service efficiency, and frees up appointments for new and acute cases.
- Digital solutions are likely to be increasingly important in enhancing PIFU, including use of ePROMs to actively monitor patients.
- Rheumatology centres should tailor their PIFU service to maximize the value for the local population.

## What is PIFU and what is the purpose of the consensus paper?

Patient initiated follow-up (PIFU) is a clinical management method which aims to optimize outpatient care delivery by providing stable patients with tools to take ownership for their own care and access clinical services as and when needed clinically. PIFU can be a valuable addition to rheumatology services where patients are managing chronic conditions. Importantly, PIFU has demonstrated comparable safety and outcomes to traditional care in numerous studies across specialties [1].

While the potential benefits of PIFU have been recognized in previous literature, the authors are conscious that establishing a new PIFU service can present unforeseen challenges. This consensus document aims to present a streamlined approach for delivering a rheumatology PIFU service and highlights how potential pitfalls can be mitigated both now and within the context of the future health service.

## Why should rheumatology services consider offering PIFU?

The gap between the demand and supply of healthcare services is steadily increasing, exacerbated by a growing workforce shortage, increasing demands on clinician time and record-breaking waiting lists [2]. Within this context, healthcare systems need to adapt to provide patients with greater ownership for managing their own care without compromising quality, clinical safety, and outcomes.

PIFU is not a novel concept but is one that is steadily gaining traction in the NHS, not only as a solution for addressing the COVID-related backlog, but also as a means for supporting patients with chronic conditions to self-manage where possible and access services when needed.

Indeed, NHS England’s 2022/23 Operational Planning guidance highlighted the potential benefits of PIFU services and suggested that a target to reduce follow-up appointments by at least 25% by March 2023 could be achieved through transferring 5% of patients onto PIFU pathways [3].

The merits of PIFU have specifically been recognized for Rheumatology patients due to the chronic nature of rheumatological conditions with the 2021 ‘Getting It Right First Time’ National Specialty Report into Rheumatology showcasing PIFU as a method to improve overall patient management by reducing unnecessary visits [4]. A 2022 systematic review by Sarah Reed and Nadia Crellin, which reviewed outcomes in single-site studies in the UK and Europe also confirmed that PIFU can be an effective model of care for rheumatology patients and can lead to a reduction in follow-up appointments as compared with standard care, with no significant difference in clinical outcomes [1].

## How can PIFU work in practice for rheumatology patients?

### Creating a PIFU pathway

The roadmap for implementing a new PIFU service can differ significantly between rheumatology centres due to variations in outpatient service maturity, clinical and operational workflows, case mix, use of digital solutions and more.

The authors agree that there is no ‘one-size-fits-all’ approach for designing and deploying a successful PIFU strategy for rheumatology patients and that it is imperative for any new patient management pathway to be designed with the unique requirements of the respective outpatient service and its local patient population needs in mind. Nevertheless, the authors have identified five core elements that need to be embedded in any successful PIFU service. These are discussed below in Fig. 2 alongside an example of an effective PIFU service model.

### 5 Key elements for developing a PIFU pathway

When designing any new healthcare pathway, it is imperative to understand the overarching goals of all key stakeholders (i.e. healthcare professionals, administrators, patients, and healthcare management). In this case, individual needs must be carefully balanced against the overall purpose of a PIFU programme, which is ultimately to improve resource allocation within the wider health service while maintaining patient safety and delivery of high-quality care and promoting patient autonomy. Although not exhaustive, considering the following five elements will help to develop a safe and effective PIFU service that meets the needs of all stakeholder groups and enable stakeholders to consider challenges that may be encountered when delivering a PIFU service.

**Figure 1.**
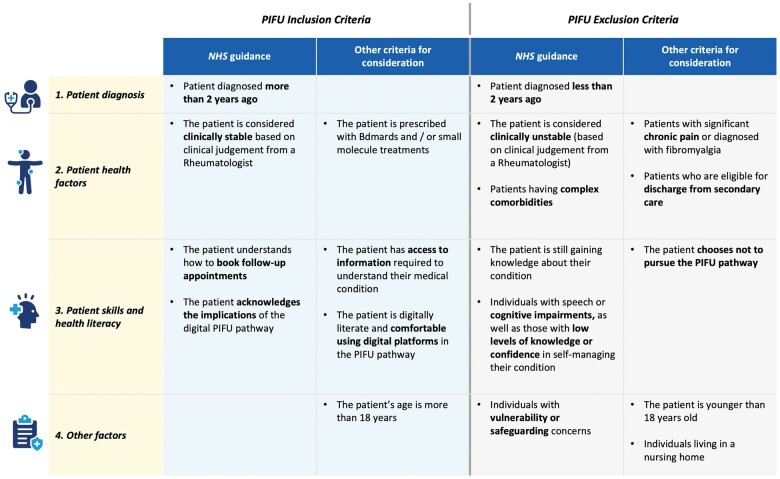
Patient identification criteria for the PIFU pathway

1. **Patient selection and offboarding criteria**
  Clear criteria must be established to identify patients eligible for PIFU. This ensures that only stable patients with a clear understanding of their health status and the confidence to manage their own care are considered. Equally, mechanisms must be present to identify when a patient is *no longer suitable* for PIFU (for instance, due to an escalation in their condition) and processes established for ensuring patients can be safely returned to a follow-up care regime if required. Regardless of eligibility, the decision to initiate a PIFU management pathway needs to be made *in collaboration* with the patient to ensure adequate buy-in and understanding. The decision to initiate PIFU management should also be communicated to the patient’s wider healthcare team (i.e. their GP and/or community care team). Figure 1 demonstrates an overview of suggested selection criteria to initiate a patient on a PIFU pathway.
2. **Patient education and engagement strategy**
  A structured patient education and engagement programme should be established to support patients to confidently engage with PIFU from day one and beyond. Patients should be educated on how to recognize, monitor, and self-manage symptoms when appropriate and be able to confidently identify ‘red flags’, such as flare symptoms, which require further discussion with their clinical team. A multi-modal approach to educational content delivery that includes face-to-face instruction, patient guides, videos and refresher courses can be beneficial in ensuring that each patient can engage with the service in their chosen manner and, in the case of digital materials, access resources at their convenience.
3. **Clinical safety and escalation framework**
  Robust clinical safety netting and escalation measures must be embedded within PIFU pathways to enable active monitoring and early identification of deterioration and ensure that patients have available mechanisms to contact their clinical team should they have any concerns. In rheumatology, PROMs completed by patients at regular intervals can be used by clinicians to support active monitoring. Patients managed through PIFU should continue to attend routine investigations, such as blood tests or imaging, through established processes.
4. **PIFU operational excellence and ways of working**
  Internal change management should not be underestimated when introducing a new care pathway. To maximize impact and minimize disruption, key internal stakeholders must be engaged at an early stage to shape the PIFU programme. Regular clinical governance review should be established to ensure ongoing evaluation of outcomes for patients on new care pathways.
  Consideration should be given to what new roles, guidelines and ways of working might need to be established to support the delivery of PIFU. For instance, centres will need to ring-fence a proportion of appointments for PIFU patients who required urgent attention due to a change in their condition and a proportion of clinical and administrative time may need to be protected for addressing queries from patients managed through a PIFU pathway.
  A standard operating procedure (SOP) specific to PIFU should be developed to support clinical and non-clinical staff understand these new PIFU-related roles and responsibilities. This should be regularly reviewed, and staff should be supported by a dedicated educational programme to enable them to confidently make decisions about patients on a PIFU pathway.
5. **Clinical governance approach**
  Clinical governance is vital to monitoring and assessing the success of a PIFU pathway. A dedicated team should review the PIFU programme at regular intervals, evaluate relevant collected data, review clinical safety aspects of the programme, including reported incidents and near-misses, and ensure overall quality control measures. The programme should include a review of patients engaged with the PIFU service and also consider the impact on the overall outpatient service more broadly.

### An example of a PIFU pathway: putting theory into practice

**Figure 2.**
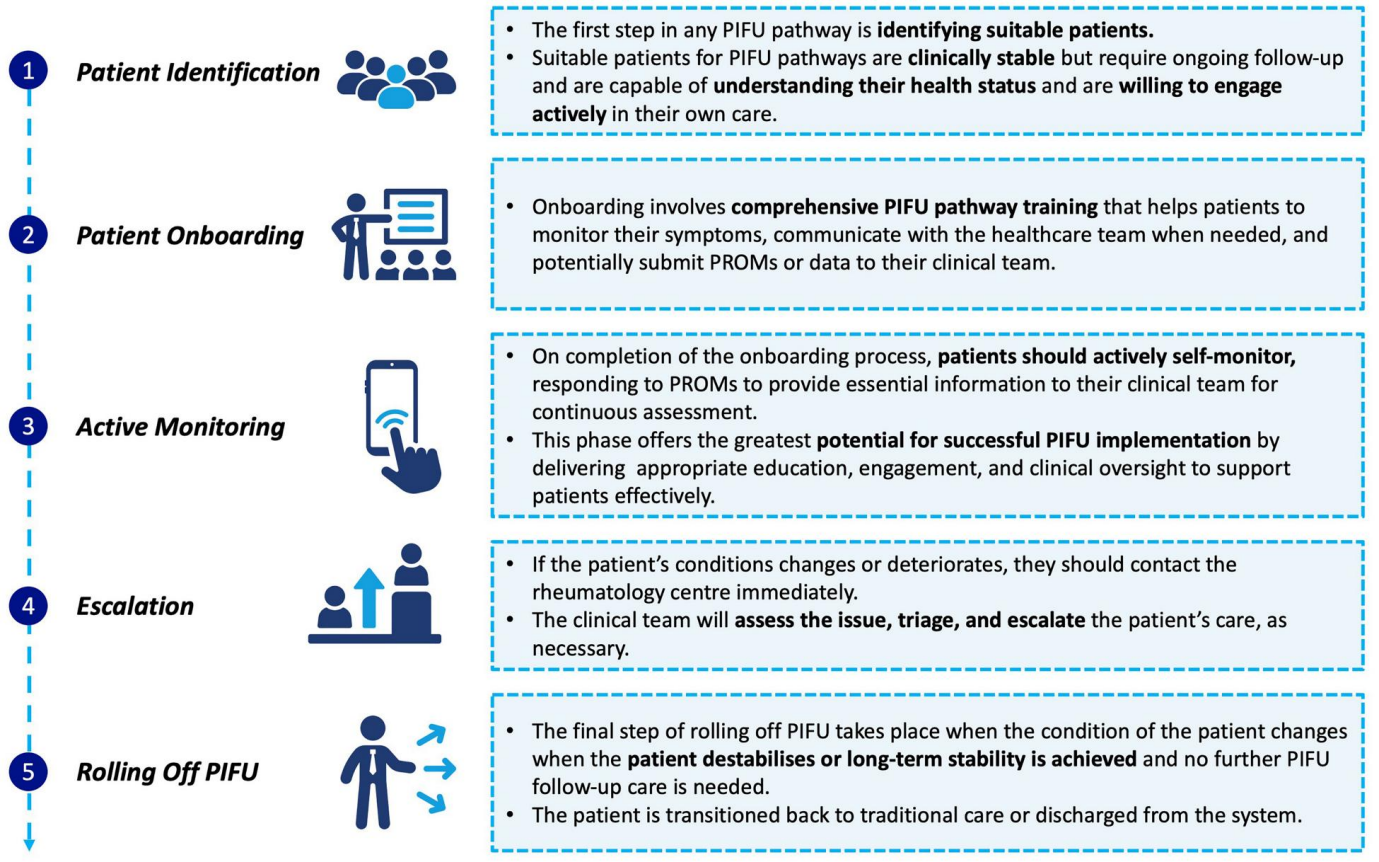
Five key phases of the PIFU pathway

- **Service overview**
  As shown in Fig. 2, a minimum of 5 core components should be included in a PIFU pathway for rheumatology patients:
  1. Patient identification
  2. Patient onboarding
  3. Active monitoring
  4. Escalation
  5. Rolling off PIFU

The delivery of each of these steps needs to be considered both individually and in the round from a logistical and clinical safety perspective to ensure that the pathway is accessible to both healthcare professionals and patients, capable of delivering the correct level of care to PIFU patients in a safe and effective manner to improve outcomes and is complementary to the overall outpatient care service.

- **Selecting the right patients for PIFU: Taking an individual approach**
  Selection criteria, as shown in Fig. 1, can be helpful tools in identifying patients where PIFU is objectively inappropriate. However, there are several more nuanced and holistic considerations that clinicians will wish to contemplate when deciding if PIFU is the right mode of delivery of care for their patient.
  - Socioeconomic status, for example, could play a role as patients in high-deprivation areas may face challenges with internet access or have unstable living conditions, impacting their ability to actively engage with the PIFU service.
  - Chronic co-morbidities, mental health issues, or a history of non-adherence may also make a clinician think differently about a patient’s eligibility.
  - Clinicians will know their patients well, having developed a relationship over the course of their chronic disease management. They will recognize patients who may not actively engage with monitoring and safety-netting systems in place. They may also recognize those patients who are either chronically anxious about their health condition, or naturally stoic, and so will not request appropriate support during times of flare.
    These wider and less objective considerations should be considered alongside set selection criteria to understand the appropriateness of PIFU for each patient on a case-by-case basis.
- **Getting active monitoring and escalation right**
  Active monitoring and escalation are arguably two of the most critical components in the PIFU pathway. The way in which these are structured will vary depending on system capabilities, workforce availability and chosen pathway delivery method (for instance, whether a digital solution is leveraged to deliver the service). Regardless of these variations, the authors have identified two key areas that should be considered:
  1. *Challenges to consider for delivering active monitoring*
    Any active monitoring strategy requires at least three elements:
    - *A methodology for delivering monitoring that accurately highlights changes in a patient’s condition in a timely way.*
    - *Availability of healthcare professionals to action any changes that might be demonstrated through the monitoring strategy.*
    - *An engaged patient.*
    Care must be taken in the selection of the methodology to ensure that it is in-line with ways of working and adequately engages the patient population.
    If, for example, a centre chooses to use ePROMs for active monitoring, they will need to consider how to manage the clinical reviewing responsibilities for completed ePROM assessments. In this case, digital systems can be useful in setting alerting thresholds for ePROM scores and question responses where only high scores or concerning responses demand a review. This can be helpful in reducing the burden on clinical staff to review all patient reported measures [5].
    Regardless of the method selected, balancing frequent patient monitoring with the potential burden of self-reporting should be considered, as constantly reminding stable patients of their medical condition can sometimes be counterproductive and lead to increased health anxiety and reduced engagement. To achieve this balance, outcome measures can be requested quarterly, biannually, and/or ad-hoc when the patient detects a change. Monitoring schemes can also be tailored based on patient preference, biologics use, clinician assessment, and the patient's ability and motivation to detect and report changes.
    An active monitoring system requires active engagement by the patient to diligently self-report their health status. Robust and efficient patient education at the time of onboarding is needed so that patients have a clear understanding of their role and feel empowered and confident in their ability to self-monitor and escalate issues appropriately. A variety of tools can be used to ensure this outcome, including face-to-face group training sessions and digital educational materials that can be referred to at the patient’s convenience. Creating approachable systems that provide a seamless user experience can also improve patient engagement.
  2. *Challenges to consider when designing an escalation strategy*
    The goal of escalation is to support patients in acute flare or distress to obtain clinical support or an appointment for expedited review with an appropriate member of staff. It is important to note that both patients and healthcare professionals should be able to identify the need for escalation. Inevitably, an escalation process can only be effective if patients are confident to escalate concerns and healthcare professionals are clear on the process to manage patients who are escalated and require clinical input.
    Appropriate availability of clinical capacity to manage patient queries and escalations is essential to the process and should be considered within the programme design. Centres should consider ringfencing appointment slots specifically for managing PIFU patient escalations and concerns. Assigning a multi-disciplinary team to manage concerns and escalations that are flagged in the first instance may allow for patients to be easily transferred to the most appropriate clinician. Regular monitoring of safety and effectiveness in any change of pathway is essential.
- **Rolling patients off PIFU safely**
  Different scenarios could lead to a patient being rolled off from the PIFU pathway. These include the patient no longer being suitable due to escalation or unstable symptoms, or a significant change in their circumstances. It could also be due to the patient being discharged back to primary care or simply, because PIFU management is no longer the patient’s preference. Often, guidelines for patient identification and enrolment are prioritized, with less emphasis placed on discharge or escalation back to a conventional care model. Guidelines should also illustrate the rolling-off criteria and recommended steps for clinical staff as patients approach the potential end of their PIFU journey. This is particularly important for patients whose situation may have escalated due to flare-ups or disease progression and need to be re-integrated into a more structured face-to-face follow-up outpatient care model.

## How can digital solutions support the delivery of PIFU?

Digital health solutions are increasingly being leveraged to support patient management and healthcare delivery. Within the context of PIFU, digital solutions can facilitate continuous active monitoring, provide a means for sustaining patient engagement and inform the development of future services.

### Sustaining patient engagement

It can be challenging to ensure that PIFU patients continue to actively take ownership for managing their condition and participate fully with the programme. Digital patient-facing platforms can encourage continuous engagement through applying behavioural science means, such as push notifications sent to a mobile or e-mail account, and potentially the introduction of gamification elements. Additional features, such as inclusion of targeted educational content and virtual community groups, can also empower patients to take more ownership for managing their condition and support self-management as appropriate.

### Leveraging data to improve services

One of the greatest advantages to using digital health solutions is the ability to collect data on individual patients as well as cohort groups and display insights in an easily accessible format for clinicians and patients alike.

Individual data can also be particularly useful in understanding patient-specific disease trends and supporting personalized health management. It may also be helpful in identifying patients who may be disengaged or no longer suitable for the service.

Regular review of data collected by digital tools should be performed by clinical governance teams and those involved with service improvement to support continuous optimization of the PIFU pathway and guard against any negative impact on patient outcomes and equity.

### Promoting health equity

It is vital that PIFU is introduced in a manner that supports the entire rheumatology population of a service and doesn't inadvertently widen existing health inequalities. Digital solutions can be particularly helpful in reducing barriers to accessing services for individuals who may have limited flexibility to attend regular appointments or are impeded from doing so due to travel costs.

However, digital solutions are not always the correct choice for every patient and care must be taken to ensure that provisions are made for individuals who may not wish to engage with digital platforms or who may struggle to use digital solutions due to availability of internet connection, reduced confidence with digital tools and lack of digital literacy.

### Creating sustainable solutions

A digital PIFU programme should be created to support changes in healthcare practices, patient populations, and technological advancements, but also scalable to allow for increasing numbers of patients to be onboarded to the pathway. This approach will ensure that PIFU continues to provide value in the long term and can evolve in response to the dynamic healthcare landscape.

## Conclusion

PIFU is increasingly gaining traction as a useful approach for allocating precious clinical resources more effectively and providing patients with the tools to take ownership for their care without compromising on clinical safety.

Within the context of rheumatology, PIFU can transform both the patient and health practitioner experience and can lead to more effective use of resources. While designing a new service can be challenging, the authors present a streamlined approach for considering the key elements of a PIFU service and exploring mitigation measures for common pitfalls.

The potential merits of digital solutions in the delivery of PIFU pathways are also explored as an option for improving patient engagement, supporting clinical decision making and building valuable data sets to gauge service performance and guide clinical governance and improvement. In the future, it may be necessary to consider how incentives & funding can drive the adoption of PIFU and enable the use of digital solutions to ensure benefits are felt equally across the country.

## Data Availability

No new data were generated or analysed in support of this research.
